# Automatic Bolus Tracking Versus Fixed Time-Delay Technique in Biphasic Multidetector Computed Tomography of the Abdomen

**DOI:** 10.5812/iranjradiol.4617

**Published:** 2014-01-30

**Authors:** Atoosa Adibi, Ali Shahbazi

**Affiliations:** 1 Department of Radiology, Isfahan University of Medical Sciences, Isfahan, Iran

**Keywords:** Multidetector Computed Tomography, Tomography, Spiral Computed

## Abstract

**Background:**

Bolus tracking can individualize time delay for the start of scans in spiral computed tomography (CT).

**Objectives:**

We compared automatic bolus tracking method with fixed time-delay technique in biphasic contrast enhancement during multidetector CT of abdomen.

**Patients and Methods:**

Adult patients referred for spiral CT of the abdomen were randomized into two groups; in group 1, the arterial and portal phases of spiral scans were started 25 s and 55 s after the start of contrast material administration; in group 2, using the automatic bolus tracking software, repetitive monitoring scans were performed within the lumen of the descending aorta as the region of interest with the threshold of starting the diagnostic scans as 60 HU. The contrast enhancement of the aorta, liver, and spleen were compared between the groups.

**Results:**

Forty-eight patients (23 males, 25 females, mean age=56.4±13.5 years) were included. The contrast enhancement of the aorta, liver, and spleen at the arterial phase was similar between the two groups (P>0.05). Regarding the portal phase, the aorta and spleen were more enhanced in the bolus-tracking group (P<0.001). The bolus tracking provided more homogeneous contrast enhancement among different patients than the fixed time-delay technique in the liver at portal phase, but not at the arterial phase.

**Conclusions:**

The automatic bolus-tracking method, results in higher contrast enhancement of the aorta and spleen at the portal phase, but has no effect on liver enhancement. However, bolus tracking is associated with reduced variability for liver enhancement among different patients.

## 1. Background

With recent advances in the technology of spiral computed tomography (CT), multi-detector CT (MDCT) imaging of the entire liver is now possible in less than 20 seconds, with two or more different perfusion phases (1). With this rapid scanning, it is important to optimize the time delay between contrast material injection and initiation of diagnostic scans, especially for the arterial phase (2). Individual variations regarding body weight, heart rate, circulation time, and cardiac impairments can influence the time window and the necessary rate and volume of the contrast material and thus may be problematic in achieving optimum contrast enhancement (3-5). Although tracking a small bolus of contrast material (10-20 mL) before the diagnostic scans can help individualize the time delay, it is a time-consuming technique (6). To overcome these limitations, a computer-assisted bolus tracking system was recently developed that automatically initiates diagnostic scans triggered by the contrast enhancement itself. Using low-dose scans (about 50 mA), this technology allows the scans to start, either manually or automatically, when the contrast enhancement rises to a predefined threshold in a region-of-interest (ROI) (7). Some studies showed that automatic bolus tracking could better individualize the time delay for initiation of diagnostic scans of the liver and also the pancreas and thus improve the degree of contrast enhancement and lesion-to-parenchyma conspicuity (8-10). However, there is still some controversy in this regard and the results of previous studies were different. Some studies recommend bolus tracking only if patients are more than 70 years old and/or have cardiovascular diseases and/or when there is no adequate antecubital vein for the injection of contrast material to reduce the additional radiation dose (11). In addition, some studies showed that with the bolus tracking method, 35% of the patients might not achieve a threshold of 50 HU above baseline by 60 seconds after injection initiation and then will require the use of a set delay (12).

## 2. Objectives

Therefore, this study was conducted to compare automatic bolus tracking with fix time-delay in biphasic contrast enhancement of the aorta, liver, and spleen during MDCT.

## 3. Patients and Methods

### 3.1. Patient Enrollment

This prospective study was conducted on 48 consecutive adult patients referred from August to November 2010 to the MDCT Unit of Alzahra Hospital (Isfahan, Iran) for contrast enhanced CT of the abdomen. Indications were uncertain liver lesions in ultrasonography (n=19), suspicious metastasis (n=15) and hepatocellular carcinoma (HCC) (n=14). Patients with heart failure were not included in the study. The Ethics Committee of Isfahan University of Medical Sciences approved the study and informed consent was obtained from all patients.

### 3.2. Imaging Technique

Patients were examined using a multi-detector CT scanner (LightSpeed VCT 64, GE Healthcare, Salt Lake City, Utah, USA). Scan parameters were: collimation 64×0.625 mm; slice thickness 5 mm; pitch 1.375; table speed/gantry rotation 55 mm; kV 140; and mA 240. An unenhanced spiral CT of the liver was performed before contrast injection. For biphasic spiral CT scans, the liver was scanned in the arterial and portal-venous phase of liver perfusion. With a power injector, 100 ml of Iopromide (Ultravist 300, Schering, Berlin, Germany) was injected in an antecubital vein at a flow rate of 4.0 mL/s.

Patients were randomized into two groups using a random table generated by the Random Allocation Software (13). In group 1, the arterial and portal-venous phase spiral scans were started 25 s and 55 s, respectively after the start of contrast material injection. In group 2, the reference scan that defines the level of the bolus tracking monitor scans was placed 1 cm below the diaphragm level on the inspiratory topogram. The automatic bolus tracking software (Snapshot Pulse/GE Healthcare, Salt Lake City, Utah, USA) was implemented at the scanner. Repetitive monitoring scans were done at one slice level during respiration at 50 mA with scan time duration of 0.5 s. The monitoring scans were started after a delay of 8 s and repeated every 2 s during quiet breathing. As soon as the contrast enhancement threshold (60 HU) was reached within the ROI (lumen of the descending aorta), the diagnostic spiral scans were initiated (8). Approximately 6-9 s (for repositioning of the table and breathing command to the patient) after exceeding the threshold level, the arterial phase spiral scan was started. The portal-venous scan was automatically started 15 s after completion of the arterial phase scan (8).

### 3.3. Assessments

In all patients, the pre-contrast hepatic density was measured by calculating the mean of six ROI measurements (area of each ROI was at least 3 cm^2^) determined at six different slice levels from the dome of the liver to its lower parts. The interval between selected cuts of liver CT scan was about 2 cm. On the post-contrast scans, the mean attenuation of normal parenchyma for each slice was assessed separately by the mean of three ROI measurements with an area of at least 3 cm^2^. Large intrahepatic vessels, liver lesions, or partial-volume effects were avoided at all ROI measurements. The maximum hepatic density in the arterial phase and the mean hepatic density in the portal-venous phase were calculated. Additionally, the pre-contrast and post-contrast density of the aorta approximately two centimeters below the diaphragm and of the spleen, at the central part of its parenchyma was determined (8).

### 3.4. Statistical Analyses

Statistical analyses were performed using SPSS software for Windows 16.0 (SPSS Inc., Chicago, Ill, USA) by independent sample t-test for unmatched samples comparing group 1 to group 2. The significance level was considered at P<0.05.

## 4. Results

**Figure 1.**
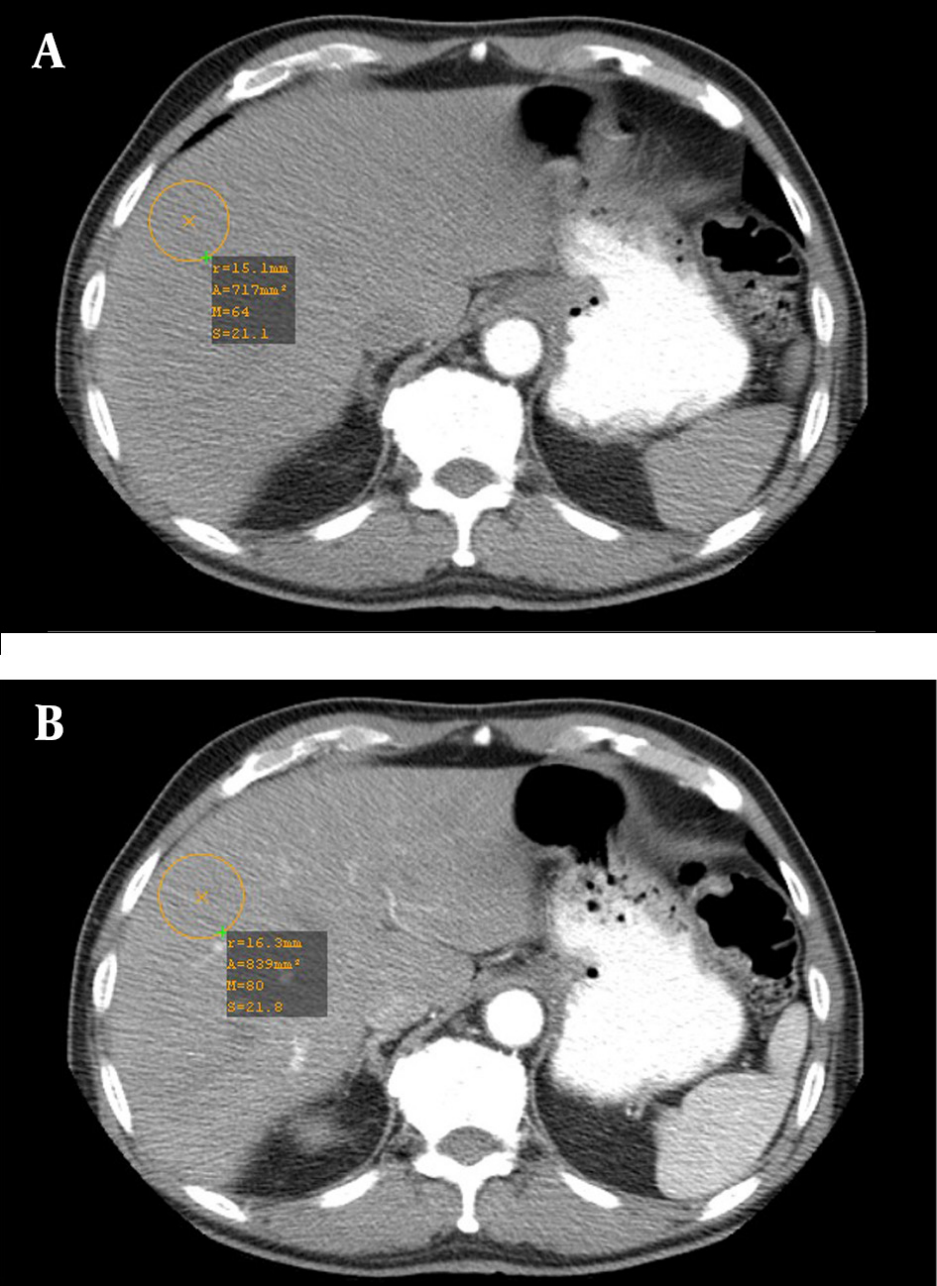
Contrast enhancement of the liver in A, arterial, and B, portal phase with fix delay times

**Figure 2.**
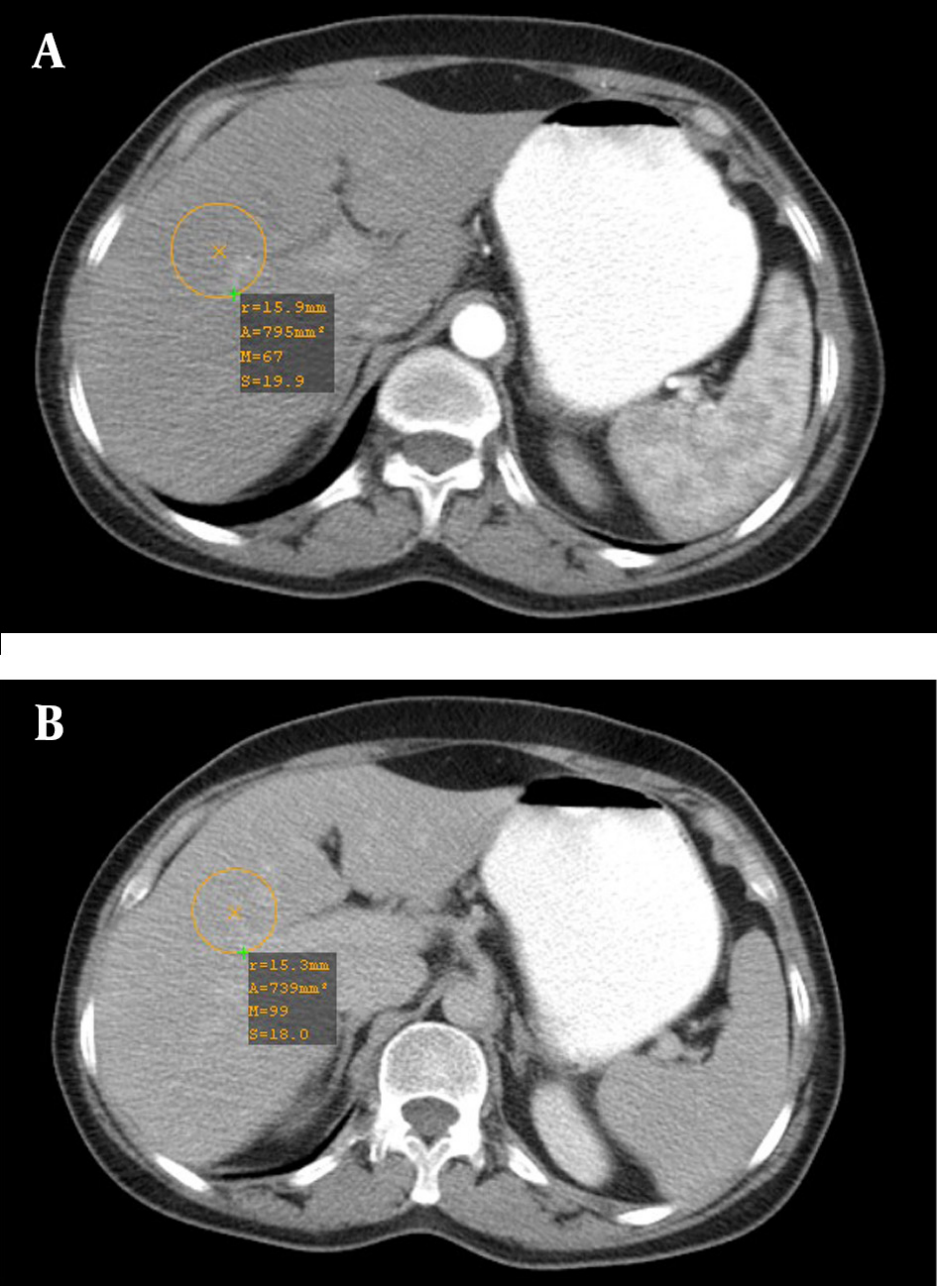
Contrast enhancement of the liver in A, arterial, and B, portal phase with bolus tracking method

During the study period, 23 males and 25 females patients with the mean age of 56.4±13.5 years were examined. The mean weight was 68.0±8.3 kg in group 1 and 70.3±8.3 kg in group 2 (P>0.05). The enhancement threshold of 60 HU in the aorta was reached in all patients 15-23 s (17.7±2.2 s) after the beginning of contrast administration. The number of monitor scans varied from 3 to 7 scans (4.5±1.1), corresponding to an additional exposure of 227.0±57.0 mAs (50 mA per slice, scan time 0.5 s). Table 1 represents pre-contrast density and enhancement (in terms of HU) of the aorta, liver, and spleen at the arterial and portal phases in the two groups. The two groups were similar regarding pre-contrast aorta, liver, and spleen density (P>0.05). There was no significant difference between the two groups in the maximum contrast enhancement of the aorta, the liver, and spleen at the arterial phase (P>0.05). Regarding the portal phase, the aorta and spleen were more enhanced in the bolus-tracking group (P<0.001). Drawing normal distribution curves, the bolus tracking provided more homogeneous contrast enhancement among different patients than the fixed time-delay technique in the liver at the portal phase, but not significantly at the arterial phase (Figures 1 and 2).

**Table 1.** Pre-Contrast Density and Enhancement (HU) of the Aorta, Liver, and Spleen at the Arterial and Portal Phases

		Fix Time-Delay ^a^(n=24)	Bolus Tracking (n=24)	P-Value
**Aorta**	Baseline	42.7±7.3	45.4±8.4	0.256
	Arterial phase enhancement	231.7±104.7	264.7±82.2	0.231
	Portal phase enhancement	126.6±37.3	271.1±121.1	<0.001
**Liver**	Baseline	58.4±10.5	57.8±8.3	0.833
	Arterial phase enhancement	5.7±4.5	5.0±5.1	0.637
	Portal phase enhancement	43.8±20.9	42.9±15.5	0.858
**Spleen**	Baseline	53.2±4.6	53.5±5.3	0.820
	Arterial phase enhancement	37.4±29.9	30.8±14.8	0.340
	Portal phase enhancement	80.2±19.1	112.2±34.7	< 0.001

^a^ Data are presented as mean±SD, Independent Sample t-Test (2-tailed) has been used for comparison of the data.

## 5. Discussion

The contrast material volume and the rate of injection are controllable factors that influence the degree of contrast enhancement in CT of the liver. However, individual variations such as body weight, heart rate, and circulation impairments are other important influencing factors that cannot be controlled or easily taken into account (3-5). Thus, a technique like automatic bolus tracking that can individualize the time delay between contrast injection and initiation of diagnostic imaging is of great value. We investigated if automatic bolus tracking, compared with fixed time-delay, can provide better contrast enhancement of the aorta, liver, and spleen during biphasic MDCT. We found that the bolus tracking method is beneficial for contrast enhancement in imagining the aorta and spleen at the portal phase, but there was no beneficial effect for this technique for liver imaging. The results of previous studies were different in this regard. Dinkel and colleagues (14) found increased hepatic enhancement at the portal phase by bolus tracking (threshold 31 HU) compared with the fixed time-delay (80 s) method. Itoh and colleagues (11) compared bolus tracking (threshold 130 HU) with fixed time-delay (30 s) in the late-arterial and portal-venous phase imaging of the liver in patients with hepatocellular carcinoma (HCC) and found no difference between the two techniques in contrast enhancement or acquisition or in the lesion-to-liver conspicuity. In another study by Mehnert et al. (9), bolus tracking (threshold 40 HU) was compared with time-delay (65 s) examinations for monophasic spiral CT of the liver andno difference was found in parenchymal enhancement between the two techniques. However, with automatic bolus tracking, a significantly higher liver-to-lesion density difference was observed (8). In another study by these investigators, on biphasic spiral CT of the liver, authors found higher maximum enhancement of the liver parenchyma in the fixed time-delay group. Investigators implied that this higher enhancement may lower the ability to delineate an arterialized lesion in the liver (9). Brodoefel and colleagues (15) compared bolus tracking (threshold of 50 HU) in terms of liver enhancement, lesion-to-liver conspicuity and inter-image variability across serial follow-up MDCTs of patients with hepatic metastases and compared it with an empirical delay of 65 s. Authors found a higher liver enhancement (about 11 UH) and conspicuity of hypo-enhancing lesions (about 20 HU) with parenchyma triggering compared with the fixed time-delay method (15).

The difference between previous studies may be related to the protocol used; threshold and the corresponding scan delay (the interval between detecting the threshold enhancement and the initiation of imaging), ROI for threshold, and contrast volume and injection rate (16, 17). In the study by Kitamura et al. (18), bolus tracking with different thresholds (120, 160, and 200 HU of the aorta) was compared with double arterial-phase (25 and 40 s) imaging for detecting hypervascular HCC. Authors found that bolus tracking with a threshold of 200 HU resulted in a higher attenuation conspicuity (42±18 HU) than with 120 HU (23±11 HU) and 160 HU (25±11 HU) and also a higher sensitivity (92.7%) than with 120 HU (72.4%) and 160HU (71.1%) (18). Sandstede et al. (19) also compared six protocols with the combination of 5- or 10s scan delays and thresholds of 50, 75, or 100 HU (in the aorta). Authors found that the 10s delay after the 75HU threshold resulted in an optimal arterial phase in most patients (defined as 20-30% of hepatic enhancement in the portal venous phase). However, compared with standard delay, bolus tracking revealed only a trend for a difference (19). The results of the study by Kim et al. (20) suggests the optimal scan delay for arterial phase images in the detection of HCC as about 14 to 30 seconds from the 100 HU threshold, while in another study by Goshima and colleagues (21), it was suggested as 10 to 15 seconds for the arterial phase and 45 to 55 seconds for the portal venous phase after the detecting threshold enhancement by 50 HU in the lower thoracic aorta 21. According to Osimani et al. (22) too early scanning results in images that are acquired before the vascular peak enhancement while too late scanning results in the increase of liver parenchyma feeding in the portal phase. Good performances were obtained with a scan delay ranging between 10 and 19 seconds from the trigger (22).

We found that bolus tracking provides more homogeneous contrast enhancement than the fixed time-delay technique among different patients. Frush and colleagues (23) in a study conducted on children also found that the bolus tracking method not only improved contrast enhancement, but also resulted in more homogeneous enhancement from the superior to the inferior levels of the liver than the fixed time-delay method. Brodoefel (15) also found that bolus tracking in the liver is associated with reduced variability for liver enhancement among different patients and across serial follow-up examinations in individual patients that permits more accurate follow-up of the patients.

The results of this study showed that the automatic bolus tracking method with 60 HU threshold at the aorta results in higher contrast enhancement of the aorta and spleen at the portal phase, but has no effects on liver enhancement. In addition, bolus tracking is associated with reduced variability for liver enhancement among different patients. Further studies are required to find the optimal protocol including threshold and the corresponding scan delay, ROI for threshold, and contrast volume and injection rate. We suggest another study with larger sample volumes in different liver pathologies comparing these post contrast methods comparing individual liver pathology separately.
